# Diagnosing and Handling Common Violations of Missing at Random

**DOI:** 10.1007/s11336-022-09896-0

**Published:** 2023-01-04

**Authors:** Feng Ji, Sophia Rabe-Hesketh, Anders Skrondal

**Affiliations:** 1 University of California, Berkeley University of Toronto, Berkeley, USA; 2 University of California, Berkeley, 2121 Berkeley Way, Berkeley, CA 94720 USA; 3 Norwegian Institute of Public Health, Oslo, Norway; 4 University of Oslo, Oslo, Norway; 5 University of California, Berkeley, Berkeley, USA

**Keywords:** data deletion, diagnostic test, graphical models, MAR, missingness mechanisms, *m*-graph, ordered factorization, structural equation models

## Abstract

Ignorable likelihood (IL) approaches are often used to handle missing data when estimating a multivariate model, such as a structural equation model. In this case, the likelihood is based on all available data, and no model is specified for the missing data mechanism. Inference proceeds via maximum likelihood or Bayesian methods, including multiple imputation without auxiliary variables. Such IL approaches are valid under a missing at random (MAR) assumption. Rabe-Hesketh and Skrondal (Ignoring non-ignorable missingness. Presidential Address at the International Meeting of the Psychometric Society, Beijing, China, 2015; Psychometrika, 2023) consider a violation of MAR where a variable *A* can affect missingness of another variable *B* also when *A* is not observed. They show that this case can be handled by discarding more data before proceeding with IL approaches. This data-deletion approach is similar to the sequential estimation of Mohan et al. (in: Advances in neural information processing systems, 2013) based on their ordered factorization theorem but is preferable for parametric models. Which kind of data-deletion or ordered factorization to employ depends on the nature of the MAR violation. In this article, we therefore propose two diagnostic tests, a likelihood-ratio test for a heteroscedastic regression model and a kernel conditional independence test. We also develop a test-based estimator that first uses diagnostic tests to determine which MAR violation appears to be present and then proceeds with the corresponding data-deletion estimator. Simulations show that the test-based estimator outperforms IL when the missing data problem is severe and performs similarly otherwise.

**Supplementary Information:**

The online version contains supplementary material available at 10.1007/s11336-022-09896-0.

A common approach for handling missing data in regression analysis (e.g., linear, logistic, multilevel) is complete-case analysis or listwise deletion. Alternatively, regression models are sometimes embedded within multivariate models in order to analyze all available data by maximum likelihood (ML) estimation, Bayesian analysis, or multiple imputation. Following Little and Zhang (2011), we use the term “ignorable likelihood” (IL) for such approaches.

Missing at random (MAR) and missing completely at random (MCAR) assumptions, originating from Rubin (1976), are often invoked as justifications for IL approaches, but these assumptions tend to be misunderstood as pointed out by Seaman et al. (2013) and Rabe-Hesketh and Skrondal (2023). When data are believed to be missing not at random (MNAR), it is commonly believed that it becomes necessary to model the missingness process explicitly. However, as demonstrated by Rabe-Hesketh and Skrondal (2015, 2023), valid inferences can often be obtained by making slight modifications to data, models, or estimators, such as data deletion followed by IL methods.

In groundbreaking work, Mohan et al. (2013) and Mohan and Pearl (2021) provide a framework for understanding missing data problems based on directed acyclic graphs (DAGs). Their DAGs, called *m*-graphs (*m* for “missing data”), represent the assumed relations among the variables of interest and include paths from these variables to missing data indicators to encode the (conditional) independence assumptions for the missing data mechanism. These representations make assumptions explicit and allow graph theory to be used to determine whether target quantities are recoverable (can be estimated consistently) and whether assumptions regarding the missingness mechanism are testable. If target quantities can be estimated consistently based on the observed data, *m*-graphs also help to derive procedures for estimating these quantities.

Following this line of research, there have been many advances in handling missing data problems, primarily in computer science. Most of this work implicitly assumes that all variables are categorical so that joint and conditional probabilities can be estimated by the corresponding sample proportions from which other target quantities can be derived. In contrast, in this paper we use *m*-graphs to derive and justify parametric inference with missing data. For concreteness, we focus on linear regression and linear structural equation modeling (SEM) and on three types of MNAR processes: (a) an explanatory variable *X* directly affects missingness of a response variable *Y* (also when *X* is not observed); (b) a response variable *Y* directly affects missingness of an explanatory variable *X* (also when *Y* is not observed); and (c) both of these MAR violations are present.

For situations (a) and (b), Rabe-Hesketh and Skrondal (2015, 2023) show that a consistent estimator can be obtained by creating more missing data followed by IL methods. They demonstrate that their data-deletion approach can be viewed as making the process MAR. Rabe-Hesketh and Skrondal (2023) demonstrate that this approach is closely related to the ordered factorization theorem of Mohan et al. (2013) but preferable for parametric models. Situation (c) can be addressed by an inverse probability weighted (IPW) estimator that we derive in Sect. 1 of this article.

In Sect. 1, we also provide a brief review of *m*-graphs and describe consistent estimators for the three types of MNAR considered. Thereafter, we use a population study in Sect. 2 to show that the estimators proposed for the above situations are actually consistent and a Monte Carlo study to investigate finite sample bias and mean squared error (MSE).

Because addressing MAR violations (a) and (b) requires different estimators, it is important to be able to diagnose which of the violations is present. We therefore propose both parametric and nonparametric diagnostic tests in Sect. 3. Simulations are used to assess the Type I error rates and power of the tests as a function of the strength of MAR violation, proportion of missing data and sample size.

In Sect. 4, we develop a novel test-based estimator that first uses diagnostic tests for specific MAR violations and then proceeds with the estimator that is valid for that violation. The finite sample performance of the proposed test-based estimator is compared with the naive IL estimator under various missing data mechanisms. Finally, we close the article with some concluding remarks.

## Missingness Mechanisms, Recoverability and Estimators

We follow the terminology and notation of Rabe-Hesketh and Skrondal (2015, 2023) in this paper. Mohan and Pearl (2021) use an *m*-graph, a type of DAG, to encode relations among multiple variables and their missingness (or selection) indicators, so that conditional independence can be inferred based on *d*-separation (e.g., Geiger et al., 1990).

**Fig. 1:**
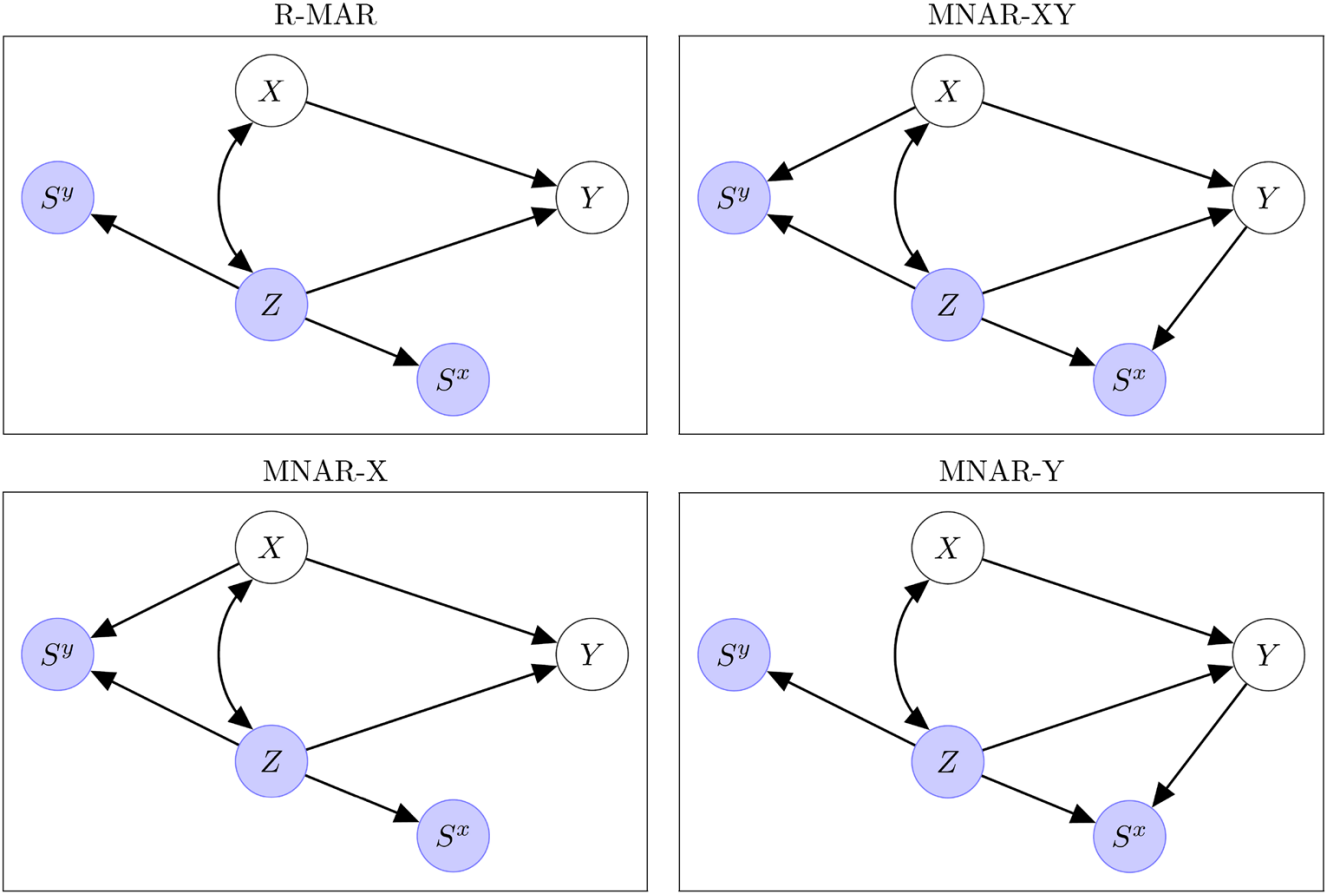
Missingness graphs (DAGs) for common missingness mechanisms.

Figure 1 shows *m*-graphs for the different types of missingness mechanisms considered in this paper. Here *Y* is the response variable and *X* and *Z* are two explanatory variables. Whereas *Z* is always observed (filled circle), *X* and *Y* can be missing (hollow circles) with corresponding selection indicators, $S^x$ and $S^y$, equal to 1 if the variable is observed and 0 if missing. In all cases, we assume that $(X_i, Z_i, Y_i, S^x_i, S^y_i)$ for unit *i* are independently and identically distributed and henceforth omit the *i* subscript for convenience. The graphs do not show “proxy” variables introduced by Mohan et al. (2013) to represent the observed data in conditional independence statements. To avoid the notation ${{\textbf{V}}}^{\textrm{obs}}$ for the subset of elements of ${{\textbf{V}}}$ that are not missing used in most of the missing-data literature, Mohan et al.’s (scalar) proxy variable $X^*$ for *X* is equal to *X* when *X* is observed and equal to some representation for missingness, such as “NA,” when *X* is missing. All variables that can be missing have such proxies, here also *Y*.

In our setting, the definition of MAR in Mohan et al. (2013) and Mohan and Pearl (2021) is . More generally, missingness cannot depend on variables that can be missing (here *X* and *Y*), given the variables that cannot be missing (here *Z*). In contrast, Rubin’s (1976) original MAR definition allows missingness to depend on ${{\textbf{V}}}^{\textrm{obs}}$, the observed elements of the variables that can be missing (and additionally, it conditions on the *realized* data and missingness indicators instead of the corresponding random variables). To emphasize that allowing missingness to depend on *X* only when it is observed is unrealistic, Rabe-Hesketh and Skrondal (2023) call Mohan and Pearl’s version of MAR “realistic MAR” (R-MAR), and we use this acronym henceforth. Rabe-Hesketh and Skrondal (2023) also relate R-MAR to Seaman et al. (2013) *everywhere* MAR and Pothoff et al. (2006) MAR+.

The *m*-graph in the top left panel of Fig. 1 represents R-MAR. For concreteness, *Y* could be income, *X* introversion and *Z* age, with income and introversion obtained from survey responses and age from an administrative database, so that age is never missing. The graph includes paths $Z \rightarrow S^x$ and $Z \rightarrow S^y$, which encode that age (*Z*) causes the missingness of the covariate (*X*) and the outcome (*Y*). For instance, senior citizens may be more reluctant to disclose their income or complete a personality questionnaire. This situation corresponds to R-MAR because *Z* is always observed.

We refer to the other situations in the figure as MNAR because R-MAR is violated. In the bottom left panel, R-MAR is violated because of one path, $X \rightarrow S^y$, where *X* is not always observed, and we call this situation MNAR-X. This path could be due to more introvert individuals having a tendency not to report their income, given their age. MNAR-Y, shown in the bottom right panel of Fig. 1, violates R-MAR because of the path $Y \rightarrow S^x$. For example, those who earn more may be less willing to provide their personal information, given age. Both MNAR-X and MNAR-Y can be handled by data deletion and standard IL based on all remaining data, as shown by Rabe-Hesketh and Skrondal (2023) and described in Sect. 1.1.

In the top right panel of Fig. 1, the paths $X \rightarrow S^y$ and $Y \rightarrow S^x$ are both present, and we call this situation MNAR-XY. This situation was not considered by Rabe-Hesketh and Skrondal (2023).

We do not consider R-MAR violations in the form of the paths $X\rightarrow S^x$ and $Y\rightarrow S^y$. While $X\rightarrow S^x$ can be handled by simply conditioning on *X* (i.e., making inferences regarding *P*(*Y*, *Z*|*X*) for the subset of data where *X* is observed), more complex solutions work in specific situations when $Y\rightarrow S^y$ (e.g., Mohan, 2018; Skrondal & Rabe-Hesketh, 2014).

Mohan and Pearl (2021) use graphs to derive whether joint and conditional distributions, such as *P*(*X*, *Z*, *Y*) and *P*(*Y*|*X*, *Z*), can be recovered (i.e., can be estimated consistently) when the missingness process is compatible with the graphs. They implicitly assume that all variables are categorical and that the distributions can be estimated from cross-tabulations. Here we assume that we have specified a correct parametric model for continuous variables, *X*, *Z* and *Y*, namely a linear SEM compatible with the graphs in Fig. 1. Our interest is in consistent estimation of the model parameters as in Mohan et al. (2018). However, we also refer to the joint distribution *P*(*X*, *Z*, *Y*) as the target when we consider the case where all parameters of the SEM are of interest, including the parameter for $\textrm{Cov}(X,Z)$, and to the conditional distribution *P*(*Y*|*X*, *Z*) when only the parameters of the regression model of *Y* on *X* and *Z* are of interest, i.e., the regression coefficients and the residual variance.

### Estimators Under MNAR-X

#### Joint Distribution

We start by showing how to recover the joint distribution *P*(*X*, *Z*, *Y*) under MNAR-X, discussed (without *Z*) in Example 1 of Mohan and Pearl (2021):

1 $$\begin{aligned} \begin{aligned} P(X, Z, Y)&= P(Z)P(X|Z)P(Y|X, Z) \\&= \underbrace{P(Z)}_{a^A}\underbrace{P(X^*|S^x = 1,Z)}_{b^A}\underbrace{P(Y^*|S^x = 1, S^y = 1,X^*, Z)}_{c^A}. \end{aligned} \end{aligned}$$

From the graph in Fig. 1, we observe that $S^x$ and *X* are independent conditional on *Z* (i.e., ), so that $P(X|Z)=P(X|S^x = 1,Z)=P(X^*|S^x=1,Z)$. We also observe that , so that $P(Y|X, Z)=P(Y| S^x = 1, S^y = 1,X, Z)=P(Y^*|S^x = 1, S^y = 1,X^*, Z)$. The last equality shows that we can apply the same estimator to the observed data *Z* and $X^*$ that we would apply if there were no missing data. To recover the joint distribution, we therefore proceed sequentially according to (1). In step $a^A$, we estimate *P*(*Z*) using all units (*Z* is never missing); in step $b^A$, we include units for which *X* is observed, $S^x=1$; and in step $c^A$, we include complete cases for which both *X* and *Y* are observed, $C=S^xS^y=1$. This is an example of Mohan et al.’s (2013) ordered factorization.

Rabe-Hesketh and Skrondal (2015, 2023) suggest a data-deletion approach briefly described here. If $S^x$ and $S^y$ satisfy MNAR-X, then the selection process for *Y* is modified by deleting *Y* when $S^x=0$, resulting in a modified selection indicator ${\dot{S}}^y$,

$$\begin{aligned} {\dot{S}}^y=\left\{ \begin{array}{cc}S^y&{}\text{ if }\ S^x=1\\ 0&{}\text{ if }\ S^x=0 \end{array}\right. . \end{aligned}$$

Now selection of *Y* depends on *X* only when *X* is observed and does not depend on any missing data, as shown by conditioning on $X^*$ (sometimes denoted $X^{\textrm{obs}}$ in the missing data literature):

2 $$\begin{aligned} \text{ M-MAR: }\ P(S^x\!\!,{\dot{S}}^y|X, Z,Y) = P(S^x|Z)P({\dot{S}}^y|S^x\!\!,X^*, Z). \end{aligned}$$

We have therefore *made* the missingness MAR, and the idea is referred to as M-MAR. If the data deletion is performed in repeated samples, the modified missingness process is everywhere MAR (as defined in Seaman et al., 2013). IL methods for all remaining data hence have the desired frequentist properties (Seaman et al., 2013).

When *X*, *Z* and *Y* follow a SEM, we call the data-deletion ML estimator SEM-X. Using the factorization in the first line of (1) to express the data-deletion log-likelihood in terms of the three densities, Rabe-Hesketh and Skrondal (2023) show that the subsets of units that contribute information on the parameters for each of these densities correspond to the subsets of units used in the sequential estimation procedure by Mohan et al. (2013). The advantage of data deletion is that we can proceed with conventional IL methods using standard software after deleting some data and obtain standard errors as a byproduct.

#### Conditional Distribution

It is evident from $c^A$ in Eq. (1) that the conditional distribution *P*(*Y*|*X*, *Z*) can be recovered from complete cases. The *m*-graph satisfies *conditional* MAR (C-MAR) as defined by Rabe-Hesketh and Skrondal (2023) because . This condition for valid inference is also discussed by Little (1992) and corresponds to Example 2 in Mohan and Pearl (2021).

### Estimators Under MNAR-Y

#### Joint Distribution

To recover the joint distribution under MNAR-Y in Fig. 1, we can again use sequential estimation, now based on the ordered factorization

3 $$\begin{aligned} \begin{aligned} P(X, Z, Y)&= P(Z)P(Y|Z)P(X|Z, Y) \\&= \underbrace{P(Z)}_{a^B}\underbrace{P(Y^*|S^y=1,Z)}_{b^B}\underbrace{P(X^*|S^x = 1, S^y = 1, Z, Y^*)}_{c^B}. \end{aligned} \end{aligned}$$

In step $a^B$, we estimate *P*(*Z*) using all units (*Z* is never missing); in step $b^B$, we include units where $S^y=1$; and in step $c^B$, we include complete cases.

After discarding values of *X* for units whose *Y* is missing, and defining the new selection indicator ${\dot{S}}^x$ as in Sect. 1.1.1 but now with *X* and *Y* interchanged, we obtain

4 $$\begin{aligned} \text{ M-MAR: }\ \ P({\dot{S}}^x\!\!,S^y|X, Z,Y) = P(S^y|Z)P({\dot{S}}^x|S^y\!\!,Z,Y^*). \end{aligned}$$

Again, the modified missingness process is everywhere MAR, and we can proceed with standard IL methods. When the assumed model for *X*, *Z* and *Y* is a SEM, we refer to the data-deletion ML estimator as SEM-Y.

#### Conditional Distribution

Unfortunately, the conditional distribution of interest *P*(*Y*|*X*, *Z*) does not appear directly in the factorization in (3). Although the conditional distributions needed for sequential estimation can be derived from the specified models for *P*(*Y*|*X*, *Z*) and *P*(*X*, *Z*), they may not be straightforward functions of the parameters of interest. Therefore, SEM-Y is highly preferable to sequential estimation here. Alternatively, an IPW estimator can be derived as shown for a more complex case in Sect. 1.3 and Appendix A.

### Estimators Under MNAR-XY

#### Joint Distribution

Mohan and Pearl (2021), Example 5, derive an expression for the joint distribution (without *Z*) that is a weighted version of the joint distribution in the complete-case sample. However, it is not clear how to apply this result unless all variables are categorical and the goal is to estimate the probabilities of the *X* by *Z* by *Y* table.

Instead, we use an estimating equation approach. Let *m*(*X*, *Z*, *Y*) be the vector of score contributions for a unit based on the joint likelihood for *X*, *Z* and *Y*. For a correctly specified model with no missing data, $E_{X,Z,Y} [m(X,Z,Y)]=0$ when evaluated at the correct parameter values. To obtain a consistent estimator of the parameters for *P*(*X*, *Z*, *Y*) using complete cases (with $C=S^xS^y=1$), we find inverse weights $\pi \equiv \pi (X,Z,Y)$ so that $E_{S^x,S^y,X,Z,Y}\left[ \frac{1}{\pi } S^xS^y m(X,Z,Y)\right] =0$ when evaluated at the correct parameter values. We show in Appendix A that

$$\begin{aligned} E\left[ \frac{1}{\pi } S^xS^y m(X,Z,Y) \right] \ =\ E_{X,Z,Y}\left[ \frac{1}{\pi } P(S^x=1| Z, Y)P(S^y=1| X, Z) m(X,Z,Y) \right] , \end{aligned}$$

where we suppressed the subscripts for the expectation when it is over the selection indicators and the variables *X*, *Z* and *Y* that are not explicitly conditioned on. Therefore, $\pi =P(S^x=1| Z, Y)P(S^y=1| X, Z)$ and both these probabilities, assumed to be positive, can be estimated in the complete-case sample by using, for instance, logistic regression. The inverses of the products of these estimates are then used as weights in pseudo maximum likelihood estimation. We call this IPW estimator SEM-XY. The estimator is also consistent under MNAR-X and MNAR-Y because these mechanisms are special cases of MNAR-XY.

#### Conditional Distribution

We now let *m*(*X*, *Z*, *Y*) be the vector of score contributions for a unit based on the likelihood of *Y* given *X* and *Z*. Without missing data, we assume that $E_{Y|X,Z,}[m(X,Z,Y)]=0$ at the correct parameter values. To estimate a model for *P*(*Y*|*X*, *Z*) using complete cases when there are missing data, we want to find $\pi \equiv \pi (X,Z,Y)$ so that $\textrm{E}_{S^x, S^y,Y|X,Z}\left[ \frac{1}{\pi } S^xS^y m(X,Z,Y)\mid X,Z\right] =0$ at the correct parameter values. Appendix A shows that we obtain

$$\begin{aligned} E\left[ \frac{1}{\pi } S^xS^y m(X, Z, Y) \mid X,Z \right] \ =\ P\left( S^y=1\mid X, Z\right) E_{Y|X,Z}\left[ \frac{1}{\pi } P\left( S^x=1\mid Z, Y\right) m(X,Z,Y) \right] . \end{aligned}$$

Assuming $P\left( S^y=1\mid X, Z\right) >0$, the estimating equation becomes $E_{Y|X,Z}\left[ \frac{1}{\pi } P\left( S^x=1\mid Z, Y\right) m(X,Z,Y)\right] =0$, so $\pi =P\left( S^x=1\mid Z, Y\right)$, also assumed to be positive. For linear regression, the pseudolikelihood estimator is weighted least squares (WLS), and we call the estimator WLS-X because the inverse weights are estimates of $P\left( S^x=1\mid Z, Y\right)$.

## Performance of Estimators

### Population Study of Asymptotic Performance

We evaluated the performance of six estimators for a simulated population-scale dataset where missingness mechanisms are compatible with the DAGs in Fig. 1. The model for *P*(*X*, *Z*, *Y*) is a SEM as described in Sect. 2.1.1. The six estimators are:

1. OLS for *P*(*Y*|*X*, *Z*): OLS with complete cases
2. SEM for *P*(*X*, *Z*, *Y*): SEM by MLE (with all available data)
3. SEM-X for *P*(*X*, *Z*, *Y*): SEM by MLE after discarding *Y* when *X* is missing
4. SEM-Y for *P*(*X*, *Z*, *Y*): SEM by MLE after discarding *X* when *Y* is missing
5. WLS-X for *P*(*Y*|*X*, *Z*): WLS with weights for selection of *X*
6. SEM-XY for *P*(*X*, *Z*, *Y*): SEM by pseudo-MLE with weights for selection of *X* and *Y*.

#### Simulation Design

For *X* and *Z*, we specify a bivariate normal distribution:

5 $$\begin{aligned} \begin{pmatrix} X\\ Z\\ \end{pmatrix}&{\mathop {\sim }\limits ^{i.i.d.}} N\begin{bmatrix} \begin{pmatrix} 0\\ 0\\ \end{pmatrix}\!\! \, , \begin{pmatrix} \psi _{XX} &{} \psi _{XZ}\\ \psi _{ZX} &{} \psi _{ZZ} \end{pmatrix} \end{bmatrix}, \end{aligned}$$

where $\psi _{XX}=\psi _{ZZ}=1$ and $\psi _{XZ}=0.5$. *Y* is simulated from the linear model

6 $$\begin{aligned} Y&= \beta _0 + \beta _XX + \beta _ZZ + \epsilon , \quad \epsilon |X,Z \sim N(0, \sigma ^2), \end{aligned}$$

where $$\beta _0 = \beta _X = \beta _Z = \sigma ^2 =1$$.

The missingness indicators $M_x=1-S^x$ and $M_y=1-S^y$ are simulated from probit models:

7 $$\begin{aligned} M_{y}^*&= \gamma _{0} +\gamma _{X}X + \gamma _{Z}Z + u_0, \quad u_0|X,Z \sim N(0, 1) \end{aligned}$$

8 $$\begin{aligned} M_{x}^*&= \alpha _{0} + \alpha _{Z}Z + \alpha _{Y}Y + u_1, \quad u_1|Z,Y \sim N(0, 1), \end{aligned}$$

where

$$\begin{aligned} 1-S^x=M_{x} = \left\{ \begin{aligned}&1,{} & {} \text {if}\ M_{x}^*> 0 \\&0,{} & {} \text {otherwise} \end{aligned} \right. \quad \quad \text{ and } \quad \quad 1-S^y=M_{y} = \left\{ \begin{aligned}&1,{} & {} \text {if}\ M_{y}^*> 0 \\&0,{} & {} \text {otherwise}. \end{aligned} \right. \end{aligned}$$

We set $\gamma _{Z} = \alpha _{Z} =1$ and express the strength of dependence represented by the paths $X\rightarrow S^y$ and $Y\rightarrow S^x$ by the partial correlations, $\rho _{M_y^*X}\equiv \textrm{Cor}(M_y^*,X|Z)$ and $\rho _{M_x^*Y}\equiv \textrm{Cor}(M_x^*,Y|X,Z)$, respectively. Appendix B gives expressions for these correlations that allow us to solve for $\gamma _X$ and $\alpha _Y$. We simulate four missingness processes defined by the four combinations of $\rho _{M_y^*X}\in \{0,0.6\}$ and $\rho _{M_x^*Y}\in \{0,0.6\}$. For MNAR-X, $\rho _{M_y^*X} = 0.6$ and $\rho _{M_x^*Y} = 0$; for MNAR-Y, $\rho _{M_y^*X} = 0$ and $\rho _{M_x^*Y} = 0.6$; for MNAR-XY, $\rho _{M_y^*X} = 0.6$ and $\rho _{M_x^*Y} = 0.6$ and for R-MAR, $\rho _{M_y^*X} = 0$ and $\rho _{M_x^*Y} = 0$. Appendix B also gives expressions for the marginal probabilities of observing *X* and *Y*, $P_x=P(S^x=1)$ and $P_y=P(S^y=1)$, which were set to 0.8 to solve for $\gamma _0$ and $\alpha _0$.

Datasets of size N = 5,000,000 were simulated with the $\texttt {R}$-package $\texttt {lavaan}$ (Rosseel, 2012) which is also used for maximum likelihood estimation of the SEM (for SEM, SEM-X, and SEM-Y). The IPW estimator was based on the correct probit models, and we implemented WLS-X with $\texttt {survey}$ (Lumley, 2019), and SEM-XY with $\texttt {ipw}$ (van der Wal & Geskus, 2011) and $\texttt {lavaan.survey}$ (Oberski, 2014). Sandwich estimators were used for the standard errors (SEs) of IPW estimators.

#### Results

Table 1 reports the point estimates of $\beta _X$ and $\beta _Z$ for all estimation methods and the estimates of $\psi _{XZ}$ for the SEM estimators. The original estimated SEs have been multiplied by 100 and are presented in parentheses. The asymptotic SEs for any sample size *m* can be obtained by multiplying the reported estimates by $\sqrt{500/m}$. Point estimates expected to be consistent are indicated by asterisks next to the estimates. SEs of the most efficient estimators are shown in bold and the percentage increase in SE compared with the smallest SE is shown in square brackets for some alternative consistent estimators for each condition. Point estimates that differ from the generating value by more than 3% are shown in italics.

We see that SEM is inconsistent when R-MAR is violated although the inconsistency of ${\widehat{\beta }}_X$ is small. In contrast, the data-deletion estimators SEM-X and SEM-Y are consistent under MNAR-X and MNAR-Y, respectively. These deletion estimators have smaller population SEs and are hence more efficient than the corresponding consistent IPW estimators, WLS-X and SEM-XY. In fact, SEM-XY is very inefficient, a general problem of IPW estimators discussed in Seaman and White (2013). SEM-X is inconsistent under MNAR-Y and, to a lesser extent, SEM-Y is inconsistent under MNAR-X. Generally, the inconsistency of ${\widehat{\beta }}_Z$ is more severe than that of ${\widehat{\beta }}_X$.

**Table 1:** Estimates and (100$\times$SE) for population study.

		MNAR-X $X \rightarrow S^y$	MNAR-Y $Y \rightarrow S^x$	MNAR-XY	R-MAR
OLS	${\widehat{\beta }}_{X}$	1.000*	*0.955*	0.963	1.000*
		**(0.064)**	(0.061)	(0.062)	(0.062) [4]
	${\widehat{\beta }}_{Z}$	0.999*	*0.902*	*0.905*	1.000*
		**(0.070)**	(0.070)	(0.067)	(0.072) [7]
SEM	${\widehat{\beta }}_{X}$	0.993	0.999	0.999	1.000*
		(0.062)	(0.060)	(0.060)	**(0.060)**
	${\widehat{\beta }}_{Z}$	0.967	1.012	*0.968*	1.000*
		(0.065)	(0.068)	(0.065)	**(0.067)**
	${\widehat{\psi }}_{XZ}$	*0.483*	*0.458*	*0.432*	0.500*
		(0.056)	(0.057)	(0.056)	**(0.057)**
SEM-X: discard *Y* when *X* is missing	${\widehat{\beta }}_{X}$	1.000*	*0.955*	*0.963*	1.000*
		**(0.064)**	(0.061)	(0.062)	(0.062) [4]
	${\widehat{\beta }}_{Z}$	0.999*	*0.902*	*0.905*	1.000*
		**(0.070)**	(0.070)	(0.067)	(0.072) [7]
	${\widehat{\psi }}_{XZ}$	0.500*	*0.392*	*0.392*	0.500*
		**(0.058)**	(0.056)	(0.056)	(0.058) [2]
SEM-Y: discard *X* when *Y* is missing	${\widehat{\beta }}_{X}$	0.998	1.000*	0.986	1.000*
		(0.062)	**(0.060)**	(0.279)	(0.060) [0]
	${\widehat{\beta }}_{Z}$	0.988	1.000*	*0.956*	1.000*
		(0.065)	**(0.068)**	(0.280)	(0.067) [0]
	${\widehat{\psi }}_{XZ}$	*0.384*	0.500*	*0.161*	0.500*
		(0.058)	**(0.063)**	(0.230)	(0.057) [0]
WLS-X: with IPW for $S^x$	${\widehat{\beta }}_{X}$	1.000*	1.010*	0.999*	1.000*
		(0.066) [3]	(1.055) [1661]	**(0.120)**	(0.064) [7]
	${\widehat{\beta }}_{Z}$	1.000*	1.001*	0.996*	1.000*
		(0.076) [9]	(0.539) [688]	**(0.202)**	(0.079) [18]
SEM-XY: with IPW for $S^x$ times IPW for $S^y$	${\widehat{\beta }}_{X}$	1.000*	1.020*	0.989*	0.999*
		(0.286) [347]	(2.387) [3884]	(0.557) [362]	(0.115) [92]
	${\widehat{\beta }}_{Z}$	0.999*	0.997*	0.992*	1.000*
		(0.660) [841]	(1.012) [1379]	(1.381) [583]	(0.279) [317]
	${\widehat{\psi }}_{XZ}$	0.484*	0.490*	0.464*	0.505*
		(0.793) [1267]	(2.724) [4238]	(1.852)	(0.605) [969]

Consistent estimators are denoted by $^*$.

Bold SE is smallest SE among estimators.

Italics show estimates that differ from generating value by at least 3%.

Square brackets show percentage increase in SE compared with smallest SE among estimators.

In contrast, OLS is consistent for MNAR-X (and R-MAR) because , the sufficient condition for recovering *P*(*Y*|*X*, *Z*), and hence consistently estimating $\beta _X$ and $\beta _Z$, using complete cases. Notably, *X* in this case can cause its own missingness (a condition not included in the simulation).

All estimators are consistent under R-MAR, with SEM and SEM-Y being more efficient than OLS. This can be understood by remembering that the maximum likelihood estimators (SEM, SEM-Y) are equivalent to the corresponding multiple imputation estimators (without auxiliary variables). Units with $S^y=1$ and $S^x=0$ can be used to impute *X* based on the imputation model *P*(*X*|*Z*, *Y*) estimated from the complete cases, and such imputations contribute to the estimation of *P*(*Y*|*X*, *Z*) and hence $\beta _X$ and $\beta _Z$. The efficiency gain is not large in the current case but could be improved by using multiple imputation with auxiliary variables.

OLS and SEM-X produce identical estimates of $\beta _X$ and $\beta _Z$ for all missingness processes because (i) without missing data, OLS estimates of regression coefficients are identical to ML estimates when the likelihood is based on *P*(*Y*|*X*, *Z*), and (ii) SEM-X uses data from complete cases only for the parameters of *P*(*Y*|*X*, *Z*), and these parameters are distinct from the parameters of *P*(*X*, *Z*) (whose estimates are also based on incomplete cases).

We note that WLS-X is consistent for *P*(*Y*|*X*, *Z*) under all missingness mechanisms considered here, and the loss in efficiency relative to the most efficient consistent alternative (when one exists) is appreciable under MNAR-Y but much less so under MNAR-X. Under MNAR-XY, WLS-X is more efficient than SEM-XY and therefore preferable if we are interested in the parameters of *P*(*Y*|*X*, *Z*) only.

### Simulation Study of Finite Sample Performance

In this section, we assess the finite-sample frequentist properties of the estimators discussed above. We limit our investigation to a comparison of SEM with each consistent estimator, i.e., SEM-X, SEM-Y and SEM-XY for MNAR-X, MNAR-Y, MNAR-XY, respectively.

#### Simulation Design

The simulation conditions are all combinations of the following factors:

1. Strength of dependence $\rho _{M_y^*X} \in \{0, 0.5, 0.9\}$.
2. Strength of dependence $\rho _{M_x^*Y} \in \{0, 0.5, 0.9\}$.
3. Marginal probability of observing *X*: $P_x \in \{0.3, 0.8\}$.
4. Marginal probability of observing *Y*: $P_y \in \{0.3, 0.8\}$.
5. Sample size: $$N \in \{200, 500, 1000\}$$.

For MNAR-X, $\rho _{M_y^*X} \in \{ 0.5, 0.9\}$ and $\rho _{M_x^*Y} = 0$; for MNAR-Y, $\rho _{M_y^*X} = 0$ and $\rho _{M_x^*Y} \in \{0.5, 0.9\}$; for MNAR-XY, $\rho _{M_y^*X} \in \{ 0.5, 0.9\}$ and $\rho _{M_x^*Y} \in \{ 0.5, 0.9\}$.

We replicate the simulation 100 times for each condition and estimate the parameters by SEM (MLE based on all available data) as well as the SEM-X, SEM-Y or SEM-XY estimator that is consistent for that condition.

#### Results

**Fig. 2:**
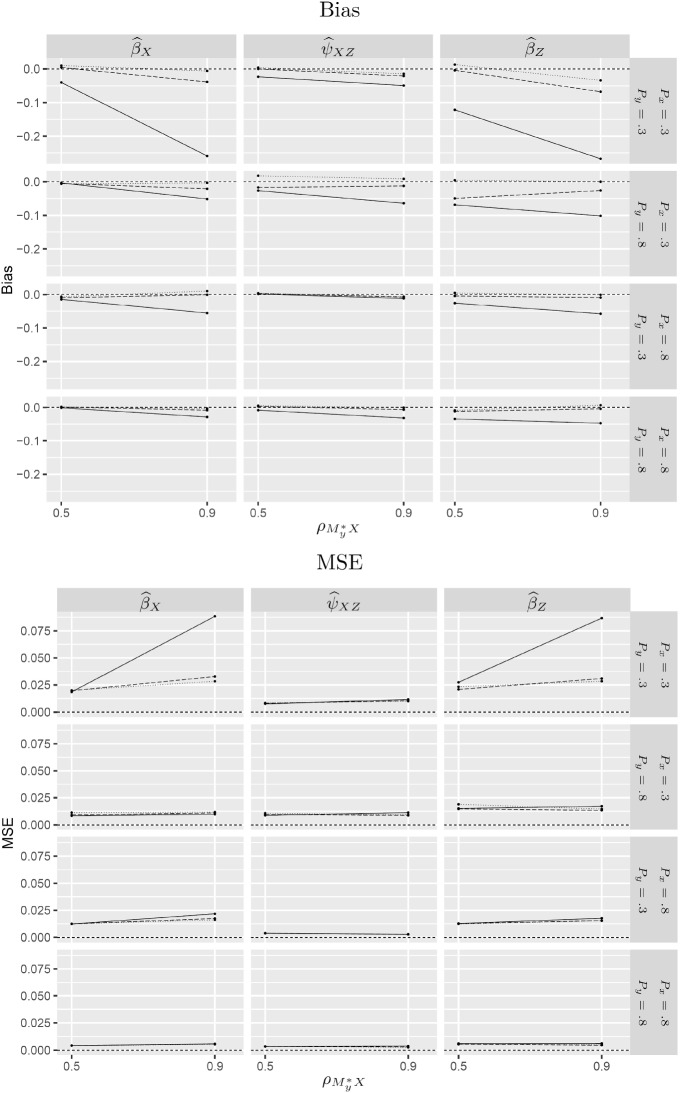
Estimated bias and mean squared error (MSE) for SEM (solid lines), SEM-X (dotted lines) and test-based (dashed lines) estimators under MNAR-X for $N=500$.

**Fig. 3:**
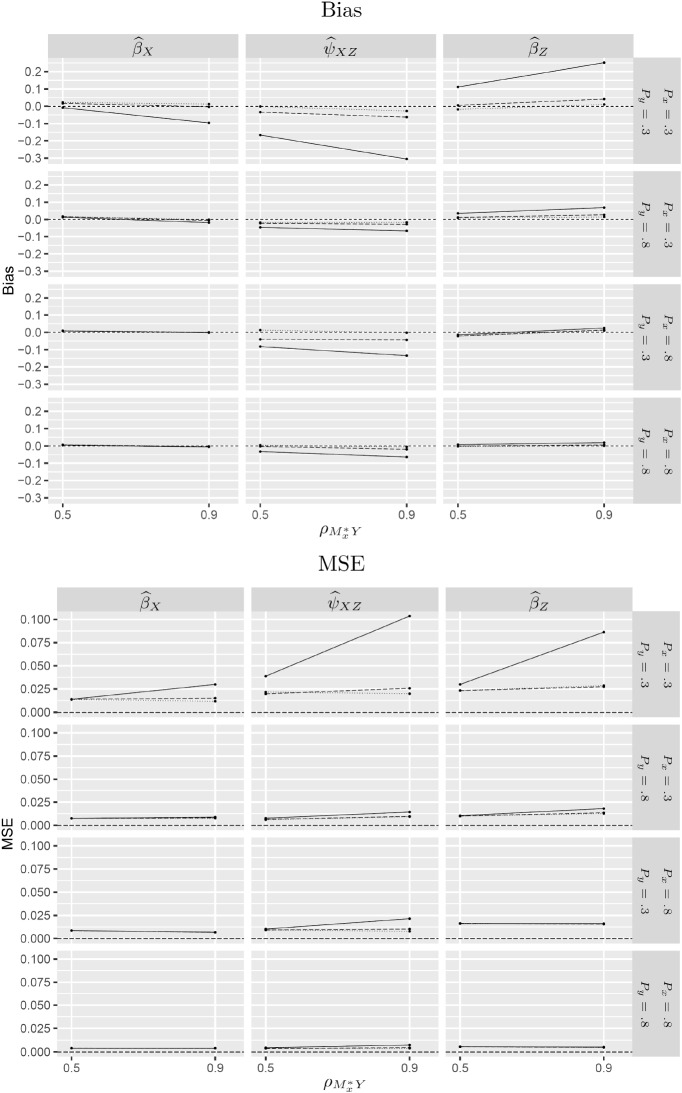
Estimated bias and mean squared error (MSE) for SEM (solid lines), SEM-Y (dotted lines) and test-based (dashed lines) estimators under MNAR-Y for $N=500$.

The estimated bias and mean squared error are reported for ${\widehat{\beta }}_X$, ${\widehat{\psi }}_{XZ}$ and ${\widehat{\beta }}_Z$ in Figs. 2 and 3 for $N=500$. Tables with these results as well as the results for $N=200$ and $N=1000$ are given in Web Appendix C (Tables 1 to 6).

**Fig. 4:**
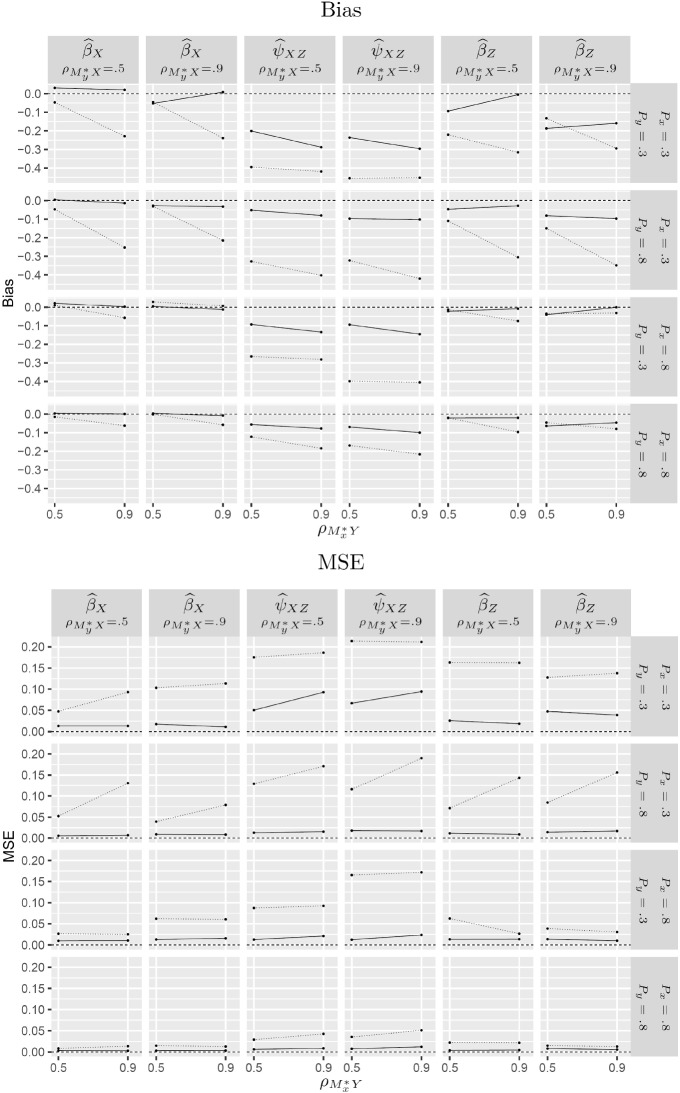
Estimated bias and mean squared error (MSE) for SEM (solid lines) and SEM-XY (dotted lines) estimators under MNAR-XY for $N=500$.

The upper panels in Figs. 2 and 3 show that the consistent estimators (SEM-X for MNAR-X and SEM-Y for MNAR-Y) are approximately unbiased whereas SEM is biased, especially when the probabilities of being observed, $P_x$ and $P_y$, are both small and the strength of dependence is large. In these conditions, the data-deletion estimators also have considerably smaller MSE than SEM in spite of the loss of data (see lower panels of Figs. 2 and 3). The tables in Web Appendix C show that these results persist for $N=1000$. However, when $N=200$, the MSE of SEM is comparable to that of SEM-X and SEM-Y.

For MNAR-XY, Fig. 4 shows that SEM-XY has greater bias and MSE than SEM for all simulation conditions although it is consistent unlike SEM.

## Diagnostic Tests for Violation of R-MAR

When missingness is believed to be MNAR, the missingness or selection process is sometimes modeled jointly with the process of interest, for example by including the outcome of interest as a covariate in the selection model (Diggle & Kenward, 1994; Hausman & Wise, 1979), allowing the error term of a probit selection model to be correlated with the error term of the linear regression model of interest (Heckman, 1979), or by including shared random effects in the selection and substantive models (Wu & Carroll, 1988). In such models, MAR can be tested by testing the null hypothesis that the parameters that represent the MAR violations are zero. However, such models and the associated tests rely on unverifiable assumptions (e.g., Molenberghs et al., 2008). Without such assumptions, MAR or Seaman et al.’s everywhere MAR are not testable because they involve statements regarding the missing data.

Little (1988) developed tests for the much stronger missing completely at random (MCAR) assumption, but these tests are of limited use for approaches that require only the weaker MAR assumption. Fortunately, as pointed out by Pothoff et al. (2006), R-MAR (which they called MAR+) can be tested. While R-MAR is a stronger assumption than MAR (or everywhere MAR), so that violation of R-MAR does not imply violation of MAR, it is difficult to imagine realistic scenarios where MAR holds and R-MAR does not. We therefore consider R-MAR tests here.

### Testability

Here we consider tests of the null hypothesis that the missingness process is R-MAR against alternative hypotheses expressed as specific violations of R-MAR. Mohan and Pearl (2021), Example 13, summarize the violations of R-MAR that are testable. Based on their work, we can test R-MAR against MNAR-X and MNAR-Y by expressing the alternative hypotheses asConditioning on $S^x = 1$ and $S^y = 1$ above ensures that the two statements involve solely observed variables and that both tests are therefore feasible.

### Likelihood Ratio Test (LRT)

We propose to use likelihood-ratio tests based on heteroscedastic regression where the groups defined by the selection indicators have different conditional expectations and variances versus a nested model that assumes equal conditional expectations and variances. Specifically, to test R-MAR against $\mathrm {H_1^{\mathrm{MNAR-X}}}$, we use the following model:

$$\begin{aligned} X^*&= \delta _0 + \delta _1Z + \delta _{2}S^y + \delta _{3}S^yZ + \epsilon ,\qquad \epsilon |Z, S^y \sim N(0, (1 - S^y)\sigma _{0}^2 + S^y\sigma _{1}^2), \end{aligned}$$

and perform a LRT to test the null hypothesis that $\delta _{2} = \delta _{3} = 0 \text { and } \sigma _{0}^2 = \sigma _{1}^2$. This null hypothesis corresponds to:

9 $$\begin{aligned} \textrm{E}(X^* | Z, S^x = 1, S^y)&= \textrm{E}(X^* | Z, S^x = 1) \\ \textrm{Var}(X^* | Z, S^x = 1, S^y)&= \textrm{Var}(X^* | Z, S^x = 1).\nonumber \end{aligned}$$

To test R-MAR against $\mathrm {H_1^{\mathrm{MNAR-Y}}}$, we use an analogous LRT based on a heteroscedastic regression of $Y^*$ on *Z*, $S^x$ and $S^xZ$, where the residual variance depends on $S^x$.

Bojinov et al. (2020) proposed a similar test but considered only conditional expectations as in (9). It seems more meaningful to test both the conditional mean and variance because this is a conditional independence test under normality. Moreover, both conditional mean and conditional variance independence are necessary (and sufficient) for estimating covariance matrices and structural equation models with missing data (Mohan et al., 2018). We anticipate that, under multivariate normality, testing the first two moments is more powerful than a generic conditional independence test such as the one described in the next subsection.

### Kernel Conditional Independence (KCI) Test

When *X*, *Y*|*Z* is bivariate normal, conditional independence is equivalent to zero partial correlation,where $\epsilon _X \equiv X - {E}(X|Z)$ and $\epsilon _Y \equiv Y - {E}(Y|Z)$. Relaxing normality, Daudin (1980) provides a characterization of conditional independence as zero partial correlation of certain functions of the random variables. Defining ${\tilde{\epsilon }}_X \equiv f(X, Z)-{E}[f(X, Z) \mid Z]$ and ${\tilde{\epsilon }}_Y \equiv g(Y, Z)-{E}[g(Y, Z) \mid Z]$, conditional independence is characterized asfor all functions *f* and *g* that satisfy ${E} [f(X, Z)^{2}]<\infty$ and ${E} [g(Y, Z)^{2}]<\infty$, respectively. We can think of ${\tilde{\epsilon }}_X$ as a “residual” function of *X*, *Z* that is uncorrelated with any function of *Z* and similarly for ${\tilde{\epsilon }}_Y$. Then conditional independence means that the residual functions ${\tilde{\epsilon }}_X$ and ${\tilde{\epsilon }}_Y$ have zero covariance.

Although conceptually simple, this characterization is hard to implement in practice. To address this problem, Zhang et al. (2012) consider a smaller class of functions from a reproducing kernel Hilbert space that makes the problem tractable and propose a kernel conditional independence (KCI) test. For a review of nonparametric conditional independence tests for continuous variables, see Li and Fan (2020).

### Power of LRT and KCI Tests

Zhang et al. (2012) showed that the KCI test has correct Type I error rates when variables are simulated from various distributions under different non-linear transformations. To assess Type I error rates and power of the two tests in the missing data setting, we perform simulations as described below. We confine our investigation to $\mathrm {H_1^{\mathrm{MNAR-Y}}}$ because the results for $\mathrm {H_1^{\mathrm{MNAR-X}}}$ should be identical due to symmetry.

#### Simulation Design

We simulate data as in Sect. 2 but with *X* being the only partially observed variable, i.e., $S^y=1$. The data-generating process is the same as in Sect. 2.1.1. The simulation conditions are:

1. Strength of dependence $\rho _{M_x^*Y} \in \{0, .1, .2, .3, .4, .5, .6, .7, .8 ,.9\}$.
2. Marginal probability of observing *X*: $P_x \in \{.2, .8\}$.
3. Sample size $$N \in \{100, 200, 500, 1000\}$$.

For each condition, 100 datasets were simulated and both tests were performed at the 5% level. All analyses were carried out in $\texttt {R}$ with the packages $\texttt {lmvar}$ (Posthuma Partners, 2019) for heteroscedastic regression and $\texttt {CondIndTests}$ (Heinze-Deml et al., 2019) for the KCI test.

#### Results

**Fig. 5:**
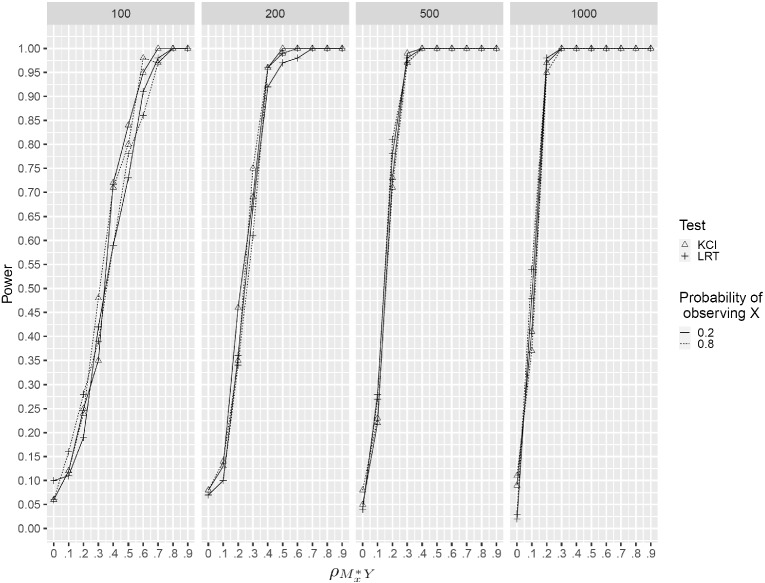
Power of test of R-MAR against $\mathrm {H_{1}^{\mathrm{MNAR-Y}}}$.

Figure 5 shows that both tests have Type I error rates at the nominal $\alpha$ level. The KCI test is comparable to LRT in terms of power in each of the simulation conditions, which is impressive given its nonparametric nature. Both tests are fairly powerful (power $\ge$ .75) for a moderate effect size ($\rho _{M_x^*Y} \ge .4$) and sample size $N \ge 200$.

## Test-Based Estimator

The simulations in Sect. 2 showed that SEM-X and SEM-Y have lower MSE than SEM if missingness is MNAR-X and MNAR-Y, respectively, especially when the strength of dependence is high and the probabilities of observing *X* and *Y* are low. It is fortunate that it is precisely in these conditions that the tests of R-MAR versus MNAR-X and MNAR-Y have high power. We therefore propose conducting these diagnostic tests and then proceeding with SEM-X if R-MAR is rejected in favor of MNAR-X and proceeding with SEM-Y if R-MAR is rejected in favor of MNAR-Y. If neither test is rejected, R-MAR is assumed to hold and SEM is used. If both tests are rejected, the process is assumed to be MNAR-XY. However, the MSE for SEM-XY is considerably greater than for SEM under MNAR-XY, so we will use SEM when both tests are rejected. These decision rules are shown in Table 2.

We use a significance level of 0.05 to avoid using the deletion estimators too readily given the good performance of SEM for small violations of R-MAR. We call the sequence of testing followed by estimation a test-based estimator. To examine the utility of the test-based estimator, we conducted a simulation study to compare the bias and MSE of the test-based estimator with SEM.

**Table 2:** Decision rules for test-based estimator, where $H_0$ is that R-MAR holds.

		
		Reject $H_0$	Not Reject $H_0$
	Reject $H_0$	MNAR-XY: SEM	MNAR-X: SEM-X
	Not Reject $H_0$	MNAR-Y: SEM-Y	R-MAR: SEM

### Simulation Study of Finite Sample Performance

The data generating process is the same as in Sect. 2.1.1, with the following conditions:

1. Strength of dependence $\rho _{M_y^*X} \in \{0, 0.5, 0.9\}$.
2. Strength of dependence $\rho _{M_x^*Y} \in \{0, 0.5, 0.9\}$.
3. Marginal probability of observing *X*: $P_x \in \{0.3, 0.8\}$.
4. Marginal probability of observing *Y*: $P_y \in \{0.3, 0.8\}$.
5. Sample size $$N \in \{200, 500, 1000\}$$.

For each combination of simulation conditions, we replicated the simulation 100 times and applied two tests (LRT and KCI, at the 5% level) and two estimators (SEM and test-based) and obtained the estimated bias and MSE for ${\hat{\beta }}_{X}$, ${\hat{\beta }}_{Z}$ and ${\hat{\psi }}_{XZ}$.

Here we report the performance of the LRT only because the KCI test performed very similarly. Figures 2 and 3 show the estimated bias and MSE for the test-based estimator (dashed lines) compared with SEM (solid lines) and the consistent estimator SEM-X for MNAR-X and SEM-Y for MNAR-Y (dotted lines). In situations where the consistent estimator performs appreciably better than SEM, the test-based estimator is not much worse than the consistent estimator. Specifically, when the marginal probabilities of being observed, $P_x$ and $P_y$, are low (first row) and the R-MAR violation is strong (large $\rho _{M_y^*x}$ for MNAR-X or large $\rho _{M_x^*y}$ for MNAR-Y), SEM performs much worse than the consistent estimator, whereas the test-based estimator performs almost as well as the consistent estimator.

**Table 3:** Bias ($\times$1000) for SEM and test-based estimator across conditions.

		$$P_x$$											
		0.3						0.8					
		$P_y$						$P_y$					
		0.3			0.8			0.3			0.8		
Method	N	${\widehat{\beta }}_{X}$	${\widehat{\beta }}_{Z}$	${\widehat{\psi }}_{XZ}$	${\widehat{\beta }}_{X}$	${\widehat{\beta }}_{Z}$	${\widehat{\psi }}_{XZ}$	${\widehat{\beta }}_{X}$	${\widehat{\beta }}_{Z}$	${\widehat{\psi }}_{XZ}$	${\widehat{\beta }}_{X}$	${\widehat{\beta }}_{Z}$	${\widehat{\psi }}_{XZ}$
SEM	200	$$-40$$	$$ -51$$	$$-173$$	$$ -20$$	$$ -44$$	$$ -56$$	$$ -9$$	$$-19$$	$$ -82$$	$$ -5$$	$$-25$$	$$-47$$
	500	−**43**	$$ -53$$	$$-175$$	$$-13$$	$$ -37$$	$$ -60$$	$$ -3$$	$$-18$$	$$ -76$$	$$ -3$$	$$-23$$	$$-49$$
	1000	$$-55$$	$$ -50$$	$$-177$$	$$-17$$	$$ -38$$	$$ -57$$	$$-15$$	$$-15$$	$$ -79$$	$$ -5$$	$$-22$$	$$-49$$
Test	200	$$ -4 $$	$$ -63$$	$$-120$$	$$ -6$$	$$ -36$$	$$ -44$$	$$ -4$$	$$-16$$	$$ -67$$	$$ -2$$	$$-21$$	$$-36$$
	500	−**0**	$$ -54$$	$$-126$$	$$ -7$$	$$ -34$$	$$ -45$$	$$ -4$$	$$-12$$	$$ -60$$	$$ -0$$	$$-18$$	$$-37$$
	1000	$$-12$$	$$ -48$$	$$-128$$	$$-11$$	$$ -33$$	$$ -40$$	$$ -7$$	$$ -8$$	$$ -62$$	$$ -2$$	$$-16$$	$$-38$$

**Table 4:** MSE ($\times 1000$) for SEM and test-based estimator across conditions.

		$$P_x$$											
		0.3						0.8					
		$P_y$						$P_y$					
		0.3			0.8			0.3			0.8		
Method	N	${\widehat{\beta }}_{X}$	${\widehat{\beta }}_{Z}$	${\widehat{\psi }}_{XZ}$	${\widehat{\beta }}_{X}$	${\widehat{\beta }}_{Z}$	${\widehat{\psi }}_{XZ}$	${\widehat{\beta }}_{X}$	${\widehat{\beta }}_{Z}$	${\widehat{\psi }}_{XZ}$	${\widehat{\beta }}_{X}$	${\widehat{\beta }}_{Z}$	${\widehat{\psi }}_{XZ}$
SEM	200	52	73	69	19	30	25	33	31	19	10	13	11
	500	24	42	53	8	13	12	12	14	12	4	5	6
	1000	18	36	50	4	8	8	7	7	11	2	3	5
Test	200	47	64	61	20	33	25	33	32	20	10	13	11
	500	16	28	41	8	13	12	12	14	11	4	5	6
	1000	8	19	37	4	8	7	6	6	9	2	3	4

Tables 3 and 4 show detailed results aggregated across the nine combinations of the three values for $\rho _{M_y^*x}$ with the three values for $\rho _{M_x^*y}$, representing R-MAR, MNAR-X, MNAR-Y and MNAR-XY. Across these missingness mechanisms, the test-based estimator had the smallest bias and MSE for most simulation conditions and comparable performance otherwise. Specifically, the test-based estimator is almost uniformly better (i.e., smaller bias and MSE) than SEM when $N = 500$ and is uniformly better when $N = 1000$ for the conditions considered. The bias reduction for the test-based estimators is evident across the simulation conditions and can be as large as $99\%$ (bold typeface in Table 3). The benefit of using the test-based estimator regarding MSE is large when the missingness of both *X* and *Y* is severe (i.e., $P_x = P_y = .3$, Table 4). Similar to the population results, ${\widehat{\beta }}_Z$ has larger bias and MSE across all simulation conditions than ${\widehat{\beta }}_X$.

The bias and MSE for each missingness mechanism separately are summarized in Web Appendix D (Tables 7 to 12). Again, the test-based estimator tends to outperform SEM when the missingness mechanisms are MNAR-X and MNAR-Y. The greater the proportion of missing data, either in X or Y, the greater the improvement in the bias and MSE, especially when the strength of dependence $\rho _{M_x^*Y}$ and $\rho _{M_y^*X}$ is high. Reassuringly, Tables 11 and 12 show that the test-based estimator performs nearly as well as SEM under R-MAR, with the larger discrepancies occurring for $N=200$.

## Concluding Remarks

We have demonstrated that the data-deletion approaches SEM-X and SEM-Y are approximately unbiased and have smaller MSE than the conventional SEM estimator for MNAR-X and MNAR-Y, respectively. We proposed tests to diagnose whether the missingness process is MNAR-X, MNAR-Y or MNAR-XY. Surprisingly, the nonparametric test had similar power to the parametric counterpart and both tests performed well for moderate departures from R-MAR. We also proposed a test-based estimator in which the choice between SEM, SEM-X and SEM-Y is determined by the diagnostic tests. This estimator outperformed the SEM estimator under MNAR-X/MNAR-Y and performed nearly as well under R-MAR.

The model considered was a simple linear SEM without latent variables, but the estimators generalize to other multivariate models such as SEMs with latent variables and/or categorical response variables.

Our missing data assumptions for consistent estimation of target quantities were confined to properties of the missingness graphs. However, in some situations, the features of the data can also be utilized to address harder questions, such as a binary outcome *Y* causing its own missingness when the explanatory variable *Z* is binary, , and there is no *X* (Mohan, 2018). In this case *P*(*Z*, *Y*) can be recovered by first estimating *P*(*Z*) and *P*(*Z*|*Y*), so that $P(Y=0)$ and $P(Y=1)$ are the only two unknowns in the two equations $P(Z=1) = P(Z=1|Y=0)P(Y=0)+ P(Z=1|Y=1)P(Y=1)$ and $P(Z=0) = P(Z=0|Y=0)P(Y=0)+ P(Z=0|Y=1)P(Y=1)$. Solving these equations gives *P*(*Y*) and hence $P(Z,Y)=P(Z|Y)P(Y)$. This estimation method will provide a unique solution only if . See also Skrondal and Rabe-Hesketh (2014) on ways to protect target parameters against inconsistency due to MNAR in mixed models for binary responses.

### Supplementary Information

Below is the link to the electronic supplementary material.

Supplementary file 1 (pdf 151 KB)

## Appendix A: Derivation of IPW Estimators

For the joint model, we now show the derivation if the IPW estimator under MNAR-XY for Sect. 1.3:

10 $$\begin{aligned} E\left[ \frac{1}{\pi } S^xS^y m(X,Z,Y) \right]&=E_{X,Z,Y}\left[ E\left( \frac{1}{\pi } S^xS^y m(X,Z,Y) \left| X, Z, Y\right. \right) \right] \nonumber \\&=E_{X,Z,Y}\left[ \frac{1}{\pi }E\left( S^xS^y\mid X, Z, Y\right) m(X,Z,Y) \right] \nonumber \\&=E_{X,Z,Y}\left[ \frac{1}{\pi } E\left( S^x\mid X, Z, Y\right) E\left( S^y\mid X, Z, Y\right) m(X,Z,Y) \right] \end{aligned}$$

11 $$\begin{aligned}&=E_{X,Z,Y}\left[ \frac{1}{\pi } E\left( S^x\mid Z, Y\right) E\left( S^y\mid X, Z\right) m(X,Z,Y) \right] \\&=E_{X,Z,Y}\left[ \frac{1}{\pi } P(S^x=1| Z, Y)P(S^y=1| X, Z) m(X,Z,Y) \right] .\nonumber \end{aligned}$$

Here (10) follows because  and (11) follows because  and . We can estimate $P(S^x=1| Z, Y)=P(S^x=1| Z, Y, S^y=1)$ in the complete-case sample. Similarly, because , we can estimate $P(S^y=1| X, Z)=P(S^y=1| X, Z, S^x=1)$.

For the conditional model, the derivation of the IPW estimator described in Sect. 1.3 is given below.

12 $$\begin{aligned} E\left[ \frac{1}{\pi } S^xS^y m(X, Z, Y) \mid X,Z \right]= & {} E_{Y|X,Z}\left[ E\left( \frac{1}{\pi } S^xS^y m(X,Z,Y) \left| X, Z, Y\right. \right) \right] \nonumber \\= & {} E_{Y|X,Z}\left[ \frac{1}{\pi } E\left( S^x\mid Z, Y\right) E\left( S^y\mid X, Z\right) m(X,Z,Y) \right] \\= & {} E\left( S^y\mid X, Z\right) E_{Y|X,Z}\left[ \frac{1}{\pi } E\left( S^x\mid Z, Y\right) m(X,Z,Y) \right] \nonumber \\= & {} P\left( S^y=1\mid X, Z\right) E_{Y|X,Z}\left[ \frac{1}{\pi } P\left( S^x=1\mid Z, Y\right) m(X,Z,Y) \right] .\nonumber \end{aligned}$$

Here (12) follows from steps analogous to those leading to (11).

## Appendix B: $\rho _{M_x^*Y|X, Z}$, $\rho _{M_y^*X|Z}$, $P_x$ and $P_y$ in terms of model parameters

For the data-generating model described in Sect. 2.1.1, we derive expressions for the strengths of dependence, $\rho _{M_x^*Y|X, Z}$, $\rho _{M_y^*X|Z}$, and probabilities, $P_x$ and $P_y$, as functions of the model parameters.

13 $$\begin{aligned}&\textrm{Var}(M_x^*|X, Z) \ = \ \alpha _Y^2\sigma ^2 + 1 \nonumber \\&\textrm{Var}(Y|X, Z) \ = \ \sigma ^2\nonumber \\&\textrm{Cov}(M_x^*, Y|X, Z) \ = \ \alpha _Y\sigma ^2\nonumber \\&\rho _{M_x^*Y|X, Z} \ = \ \frac{\alpha _Y\sigma ^2}{\sqrt{\alpha _Y^2\sigma ^2 + 1 } \sqrt{\sigma ^2}} \end{aligned}$$

14 $$\begin{aligned}&\textrm{Var}(M_y^*|Z) \ = \ \gamma _{X}^2\psi _{XX} + 1\nonumber \\&\textrm{Var}(X|Z) \ = \ (1 - \frac{\psi _{XZ}^2}{\psi _{XX}\psi _{ZZ}})\psi _{XX}\nonumber \\&\textrm{Cov}(M_y^*, X|Z) \ = \ \gamma _{X}\psi _{XX}\nonumber \\&\rho _{M_y^*X|Z} \ = \ \frac{\gamma _{X}\psi _{XX}}{\sqrt{\gamma _{X}^2\psi _{XX} + 1 } \sqrt{(1 - \frac{\psi _{XZ}^2}{\psi _{XX}\psi _{ZZ}})\psi _{XX}}} \end{aligned}$$

15 $$\begin{aligned}&\textrm{E}(M_x^*) \ = \ \alpha _0 + \alpha _Y\beta _0\nonumber \\&\textrm{Var}(M_x^*) = 1 + \alpha _Z^2\psi _{ZZ} + \alpha _Y^2( \beta _X^2\psi _{XX}+\beta _Z^2\psi _{ZZ}+2\beta _X\beta _Z\psi _{XZ} +\sigma ^2) + 2\alpha _Z\alpha _Y(\beta _X\psi _{XZ} + \beta _Z\psi _{ZZ})\nonumber \\&P_x = P(S^x = 1) = P(M_x^* \le 0)\nonumber \\&= \Phi (\frac{ -(\alpha _0 + \alpha _Y\beta _0)}{ \sqrt{1 + \alpha _Z^2\psi _{ZZ} + \alpha _Y^2( \beta _X^2\psi _{XX}+\beta _Z\psi _{ZZ}+2\beta _X\beta _Z\psi _{XZ} +\sigma ^2) + 2\alpha _Z\alpha _Y(\beta _X\psi _{XZ} + \beta _Z\psi _{ZZ})}}) \end{aligned}$$

16 $$\begin{aligned}&\textrm{E}(M_y^*) \ = \ \gamma _0 + \gamma _Y\beta _0\nonumber \\&\textrm{Var}(M_y^*) \ = \ \gamma _{X}^2\psi _{XX}+ \gamma _{Z}^2\psi _{ZZ} + 2\gamma _{Z}\gamma _{X}\psi _{XZ} + 1\nonumber \\&P_y = P(S^y = 1) = P(M_y^* \le 0) \ = \ \Phi (\frac{-(\gamma _0 + \gamma _Y\beta _0)}{\sqrt{\gamma _{X}^2\psi _{XX}+ \gamma _{Z}^2\psi _{ZZ} + 2\gamma _{Z}\gamma _{X}\psi _{XZ} + 1}}) \end{aligned}$$

All parameters, except $\alpha _0$, $\gamma _0$, $\alpha _Y$ and $\gamma _X$, are fixed. To achieve desired strengths of dependence, Eqs. (13) and (14) are solved for $\alpha _Y$ and $\gamma _X$, respectively. To achieve desired marginal probabilities of observing *X* and *Y*, Eqs. (15), (16) are then solved for $\alpha _0$ and $\gamma _0$, respectively.
