# Supplementary material for: Diagnosing and Handling Common Violations of Missing at Random
Source: Psychometrika. 2023 Jan 4;88(4):1123–43. doi: 10.1007/s11336-022-09896-0 (PMC10656344; doi:10.1007/s11336-022-09896-0)
Supplement: Supplementary file 1 — (pdf 151 KB) [file 11336_2022_9896_MOESM1_ESM.pdf]

WEB APPENDIX

DIAGNOSING AND HANDLING COMMON VIOLATIONS OF MISSING AT RANDOM

PSYCHOMETRIKA, 2023. https://doi.org/10.1007/s11336-022-09896-0

Feng Ji

UNIVERSITY OF CALIFORNIA, BERKELEY

UNIVERSITY OF TORONTO, CANADA

Sophia Rabe-Hesketh

UNIVERSITY OF CALIFORNIA, BERKELEY

Anders Skrondal

NORWEGIAN INSTITUTE OF PUBLIC HEALTH

UNIVERSITY OF OSLO

UNIVERSITY OF CALIFORNIA, BERKELEY

Correspondence should be made to Sophia Rabe-Hesketh, University of California, Berkeley, 2121 Berkeley Way, CA 94720, USA. Email: sophiarh@berkeley.edu

DIAGNOSING AND HANDLING COMMON VIOLATIONS OF MISSING AT RANDOM

Web Appendix C: Performance of SEM, SEM-X, SEM-Y, and SEM-XY

Table 1: Bias ($\times$ 1000) of SEM and SEM-X under MNAR-X

			P_x											
			0.3						0.8					
			P_y						P_y					
			0.3			0.8			0.3			0.8		
Methods	$\rho_{M_y^* X}$	N	$\widehat{\beta}_X$	$\widehat{\beta}_Z$	$\widehat{\psi}_{XZ}$	$\widehat{\beta}_X$	$\widehat{\beta}_Z$	$\widehat{\psi}_{XZ}$	$\widehat{\beta}_X$	$\widehat{\beta}_Z$	$\widehat{\psi}_{XZ}$	$\widehat{\beta}_X$	$\widehat{\beta}_Z$	$\widehat{\psi}_{XZ}$
SEM	0.5	200	−30	−188	−27	−13	−31	−68	−15	−8	−5	−19	−18	−10
		500	−39	−121	−23	−3	−68	−26	−14	−25	−1	−1	−34	−8
		1000	−37	−144	−33	−8	−55	−43	−28	−24	0	−13	−22	−14
	0.9	200	−268	−239	−26	−39	−93	−66	−78	−48	−4	−22	−69	−15
		500	−258	−267	−49	−51	−101	−64	−55	−57	−11	−28	−47	−31
		1000	−282	−246	−32	−47	−96	−78	−74	−76	−6	−20	−49	−24
SEM-X	0.5	200	−18	−53	−1	−21	−24	−39	−9	−35	−7	−15	−15	−7
		500	−9	−12	−3	−6	−4	−17	−6	−4	−3	−1	−9	−4
		1000	−12	−4	−6	−0	−9	−0	−20	−4	−1	−8	−5	−0
	0.9	200	−6	−34	−6	−4	−18	−10	−19	−5	0	−3	−23	−13
		500	−5	−33	−14	−3	0	−8	−10	−0	−6	−3	−6	0
		1000	−21	0	−4	−3	−13	−3	−8	−13	0	−2	−4	−5

Table 2: MSE (× 1000) of SEM and SEM-X under MNAR-X

			P_x											
			0.3						0.8					
			P_y						P_y					
			0.3			0.8			0.3			0.8		
Methods	$\rho_{M_y^* X}$	N	$\widehat{\beta}_X$	$\widehat{\beta}_Z$	$\widehat{\psi}_{XZ}$	$\widehat{\beta}_X$	$\widehat{\beta}_Z$	$\widehat{\psi}_{XZ}$	$\widehat{\beta}_X$	$\widehat{\beta}_Z$	$\widehat{\psi}_{XZ}$	$\widehat{\beta}_X$	$\widehat{\beta}_Z$	$\widehat{\psi}_{XZ}$
SEM	0.5	200	32	83	24	26	23	19	31	36	10	9	14	5
		500	18	27	7	8	15	8	12	12	3	4	6	3
		1000	6	29	5	3	6	5	6	6	1	2	2	1
	0.9	200	150	110	27	16	24	18	46	28	10	12	14	8
		500	88	87	11	9	17	11	21	17	2	5	6	3
		1000	92	70	4	6	12	8	15	10	1	2	4	2
SEM-X	0.5	200	48	91	25	31	36	18	32	40	10	9	15	5
		500	20	23	8	11	18	10	12	12	3	4	5	3
		1000	7	14	3	5	6	4	6	6	1	2	2	1
	0.9	200	103	99	25	21	31	23	38	29	10	13	11	8
		500	28	28	10	11	14	9	16	15	2	5	5	2
		1000	15	16	4	6	8	3	9	5	1	2	2	1

Table 3: Bias (× 1000) of SEM and SEM-Y under MNAR-Y

			P_x											
			0.3						0.8					
			P_y						P_y					
			0.3			0.8			0.3			0.8		
Methods	$\rho_{M_x^* Y}$	N	$\widehat{\beta}_X$	$\widehat{\beta}_Z$	$\widehat{\psi}_{XZ}$	$\widehat{\beta}_X$	$\widehat{\beta}_Z$	$\widehat{\psi}_{XZ}$	$\widehat{\beta}_X$	$\widehat{\beta}_Z$	$\widehat{\psi}_{XZ}$	$\widehat{\beta}_X$	$\widehat{\beta}_Z$	$\widehat{\psi}_{XZ}$
SEM	0.5	200	−19	−108	−176	−3	−27	−33	−3	−36	−99	−5	−6	−30
		500	−8	−111	−166	−12	−35	−45	−7	−14	−82	−4	−7	−32
		1000	−47	−140	−173	−3	−19	−25	−3	−15	−94	−2	−10	−31
	0.9	200	−92	−275	−292	−16	−44	−47	−27	−9	−137	−11	−18	−62
		500	−96	−251	−305	−18	−68	−65	−1	−24	−134	−5	−19	−63
		1000	−107	−256	−299	−7	−45	−46	−0	−17	−129	−4	−22	−65
SEM-Y	0.5	200	−49	−15	−24	−4	−2	−3	−5	−47	−4	−3	−3	−6
		500	−23	−17	−1	−17	−8	−17	−8	−22	−13	−5	−2	−3
		1000	−16	−11	−2	−2	−8	−2	−2	−7	−8	−3	0	−7
	0.9	200	−14	−12	−1	−4	0	−3	−33	−27	−3	−8	−2	−12
		500	−13	−10	−27	−1	−14	−16	−0	−10	−2	−2	−0	−5
		1000	−3	−6	−10	−6	−10	−7	−3	−0	−21	−1	−4	−8

Table 4: MSE ($\times$ 1000) of SEM and SEM-Y under MNAR-Y

			P_x											
			0.3						0.8					
			P_y						P_y					
			0.3			0.8			0.3			0.8		
Methods	$\rho_{M_x^* Y}$	N	$\widehat{\beta}_X$	$\widehat{\beta}_Z$	$\widehat{\psi}_{XZ}$	$\widehat{\beta}_X$	$\widehat{\beta}_Z$	$\widehat{\psi}_{XZ}$	$\widehat{\beta}_X$	$\widehat{\beta}_Z$	$\widehat{\psi}_{XZ}$	$\widehat{\beta}_X$	$\widehat{\beta}_Z$	$\widehat{\psi}_{XZ}$
SEM	0.5	200	32	60	61	14	30	24	31	33	18	10	13	9
		500	13	30	38	7	10	7	8	16	10	4	5	4
		1000	8	28	35	3	5	5	5	6	10	1	2	2
	0.9	200	64	127	112	19	36	32	29	35	27	8	13	10
		500	29	86	103	8	18	14	6	16	21	4	5	7
		1000	22	80	94	3	8	6	4	7	18	1	2	5
SEM-Y	0.5	200	35	53	55	13	30	22	31	35	27	10	13	10
		500	13	23	21	7	9	6	8	16	9	3	5	4
		1000	5	10	7	2	4	4	5	6	4	1	2	1
	0.9	200	42	60	51	18	35	32	28	34	31	8	13	9
		500	11	29	19	7	12	9	6	15	7	3	4	3
		1000	6	11	8	2	6	4	3	6	4	1	2	2

Table 5: Bias (× 1000) of SEM and SEM-XY under MNAR-XY

				P_x											
				0.3						0.8					
				P_y						P_y					
				0.3			0.8			0.3			0.8		
Methods	$\rho_{M_y^* X}$	$\rho_{M_x^* Y}$	N	$\widehat{\beta}_X$	$\widehat{\beta}_Z$	$\widehat{\psi}_{XZ}$	$\widehat{\beta}_X$	$\widehat{\beta}_Z$	$\widehat{\psi}_{XZ}$	$\widehat{\beta}_X$	$\widehat{\beta}_Z$	$\widehat{\psi}_{XZ}$	$\widehat{\beta}_X$	$\widehat{\beta}_Z$	$\widehat{\psi}_{XZ}$
SEM	0.5	0.5	200	−30	−49	−195	−16	−50	−70	−4	−39	−101	−9	−22	−57
			500	−31	−93	−201	−4	−47	−52	−21	−21	−93	−3	−21	−56
			1000	−15	−70	−207	−6	−44	−57	−9	−25	−93	−3	−34	−53
		0.9	200	−0	−10	−281	−13	−49	−62	−4	−15	−141	0	−15	−74
			500	−20	−4	−288	−14	−28	−80	−2	−8	−134	−0	−20	−77
			1000	−1	−6	−304	−0	−33	−65	−4	−15	−143	−3	−17	−73
	0.9	0.5	200	−42	−177	−266	−39	−107	−87	−23	−21	−92	−11	−63	−72
			500	−52	−186	−236	−27	−81	−97	−4	−40	−94	−3	−64	−69
			1000	−38	−201	−238	−39	−92	−102	−3	−30	−95	−7	−54	−81
		0.9	200	−19	−165	−291	−35	−114	−89	−3	−1	−152	−9	−65	−104
			500	−8	−158	−296	−32	−97	−102	−12	0	−145	−7	−46	−99
			1000	−13	−177	−306	−43	−90	−88	−28	−8	−144	−4	−56	−96
SEM-XY	0.5	0.5	200	−21	−140	−382	−75	−185	−344	−49	−56	−315	−14	−43	−133
			500	−46	−220	−394	−47	−110	−327	−10	−13	−265	−14	−20	−122
			1000	−81	−164	−400	−53	−128	−288	−1	−51	−253	−6	−12	−81
		0.9	200	−238	−352	−435	−240	−341	−403	−61	−106	−303	−69	−84	−198
			500	−229	−315	−418	−252	−305	−402	−57	−74	−281	−62	−96	−184
			1000	−236	−337	−424	−243	−339	−393	−41	−75	−270	−57	−90	−174
	0.9	0.5	200	−95	−96	−454	−62	−148	−335	−25	−24	−415	−23	−24	−182
			500	−45	−132	−455	−31	−149	−322	−29	−35	−398	−1	−46	−169
			1000	−51	−111	−438	−59	−122	−316	−20	−17	−394	−11	−29	−139
		0.9	200	−202	−311	−462	−224	−388	−405	−18	−26	−405	−53	−88	−232
			500	−238	−294	−452	−214	−348	−420	−7	−31	−405	−57	−80	−216
			1000	−273	−265	−456	−178	−338	−387	−63	−31	−378	−55	−87	−204

Table 6: MSE (× 1000) of SEM and SEM-XY under MNAR-XY

				P_x											
				0.3						0.8					
				P_y						P_y					
				0.3			0.8			0.3			0.8		
Methods	$\rho_{M_y^* X}$	$\rho_{M_x^* Y}$	N	$\widehat{\beta}_X$	$\widehat{\beta}_Z$	$\widehat{\psi}_{XZ}$	$\widehat{\beta}_X$	$\widehat{\beta}_Z$	$\widehat{\psi}_{XZ}$	$\widehat{\beta}_X$	$\widehat{\beta}_Z$	$\widehat{\psi}_{XZ}$	$\widehat{\beta}_X$	$\widehat{\beta}_Z$	$\widehat{\psi}_{XZ}$
SEM	0.5	0.5	200	38	38	63	23	25	23	21	31	18	11	12	10
			500	13	26	50	5	11	12	9	13	13	3	4	6
			1000	6	13	47	3	7	8	3	5	11	1	3	4
		0.9	200	46	55	99	14	32	29	30	26	27	9	10	14
			500	13	18	93	6	8	15	10	13	21	3	4	8
			1000	7	7	97	3	5	8	4	7	22	1	2	6
	0.9	0.5	200	42	72	95	18	33	28	36	37	18	12	14	12
			500	17	47	67	8	14	17	12	13	12	3	8	7
			1000	6	46	61	4	11	13	8	6	11	2	4	8
		0.9	200	26	61	107	19	42	31	44	23	31	8	13	19
			500	11	39	94	8	16	16	15	9	24	3	5	12
			1000	6	37	97	5	12	12	8	5	22	1	4	10
SEM-XY	0.5	0.5	200	79	179	159	65	133	135	46	64	119	20	31	35
			500	47	163	175	52	71	129	26	62	87	8	22	29
			1000	55	115	172	28	77	96	17	36	75	12	17	24
		0.9	200	129	208	205	103	172	174	42	77	109	20	22	47
			500	93	162	186	130	143	171	25	26	92	13	21	42
			1000	82	146	186	84	142	164	15	30	82	9	13	38
	0.9	0.5	200	161	109	222	62	120	128	105	72	188	22	27	47
			500	103	127	214	39	84	116	62	38	165	15	15	35
			1000	62	64	199	32	64	109	49	30	165	11	7	30
		0.9	200	108	169	224	89	213	173	96	53	174	17	22	64
			500	113	137	212	78	156	190	60	30	172	13	13	50
			1000	131	105	214	53	137	156	70	29	149	9	12	46

Web Appendix D: Performance of test-based estimator

Table 7: Bias (× 1000) of SEM and test-based estimator (based on LRT) under MNAR-X.

			P_x											
			0.3						0.8					
			P_y						P_y					
			0.3			0.8			0.3			0.8		
Methods	$\rho_{M_y^* X}$	N	$\widehat{\beta}_X$	$\widehat{\beta}_Z$	$\widehat{\psi}_{XZ}$	$\widehat{\beta}_X$	$\widehat{\beta}_Z$	$\widehat{\psi}_{XZ}$	$\widehat{\beta}_X$	$\widehat{\beta}_Z$	$\widehat{\psi}_{XZ}$	$\widehat{\beta}_X$	$\widehat{\beta}_Z$	$\widehat{\psi}_{XZ}$
SEM	0.5	200	−30	−188	−27	−13	−31	−68	−14	−8	−5	−19	−18	−10
		500	−39	−121	−23	−3	−68	−26	−14	−25	−1	−1	−34	−8
		1000	−37	−144	−33	−8	−55	−43	−28	−24	0	−13	−21	−14
	0.9	200	−268	−239	−26	−39	−93	−65	−78	−47	−4	−21	−69	−14
		500	−258	−267	−49	−51	−101	−64	−55	−57	−11	−28	−47	−31
		1000	−282	−246	−32	−47	−96	−78	−74	−76	−6	−20	−49	−24
Test	0.5	200	−8	−126	−19	−14	−11	−59	−9	−25	−5	−17	−6	0
		500	−4	−3	−0	−4	−49	−17	−9	−4	−3	−1	−11	−3
		1000	−8	−23	−10	−1	−10	−13	−20	−1	−1	−8	−3	−1
	0.9	200	−34	−55	0	−3	−9	−7	−39	−13	−1	−1	−29	−9
		500	−38	−67	−20	−21	−26	−12	0	−9	−7	−8	−4	−6
		1000	−57	−36	−2	−15	−22	−27	−15	−22	−1	−1	−7	−1

Table 8: MSE (× 1000) of SEM and test-based estimator (based on LRT) under MNAR-X.

			P_x											
			0.3						0.8					
			P_y						P_y					
			0.3			0.8			0.3			0.8		
Methods	$\rho_{M_y^* X}$	N	$\widehat{\beta}_X$	$\widehat{\beta}_Z$	$\widehat{\psi}_{XZ}$	$\widehat{\beta}_X$	$\widehat{\beta}_Z$	$\widehat{\psi}_{XZ}$	$\widehat{\beta}_X$	$\widehat{\beta}_Z$	$\widehat{\psi}_{XZ}$	$\widehat{\beta}_X$	$\widehat{\beta}_Z$	$\widehat{\psi}_{XZ}$
SEM	0.5	200	32	83	24	26	23	19	31	36	10	9	14	5
		500	18	27	7	8	15	8	12	12	3	4	6	3
		1000	6	29	5	3	6	5	6	6	1	2	2	1
	0.9	200	150	110	27	16	24	18	46	28	10	12	14	8
		500	88	87	11	9	17	11	21	17	2	5	6	3
		1000	92	70	4	6	12	8	15	10	1	2	4	2
Test	0.5	200	36	84	24	29	28	18	31	37	10	9	15	6
		500	19	21	8	9	14	9	12	12	3	4	5	3
		1000	7	15	4	4	6	3	6	6	1	2	2	1
	0.9	200	117	87	26	18	27	18	39	28	10	13	10	8
		500	32	31	10	11	13	8	17	15	2	5	4	3
		1000	26	22	4	5	7	5	10	6	1	2	2	1

Table 9: Bias (× 1000) of SEM and test-based estimator (based on LRT) under MNAR-Y.

			P_x											
			0.3						0.8					
			P_y						P_y					
			0.3			0.8			0.3			0.8		
Methods	$\rho_{M_x^* Y}$	N	$\widehat{\beta}_X$	$\widehat{\beta}_Z$	$\widehat{\psi}_{XZ}$	$\widehat{\beta}_X$	$\widehat{\beta}_Z$	$\widehat{\psi}_{XZ}$	$\widehat{\beta}_X$	$\widehat{\beta}_Z$	$\widehat{\psi}_{XZ}$	$\widehat{\beta}_X$	$\widehat{\beta}_Z$	$\widehat{\psi}_{XZ}$
SEM	0.5	200	−18	−108	−176	−3	−27	−33	−3	−36	−99	−4	−6	−30
		500	−8	−111	−166	−12	−35	−45	−7	−14	−82	−4	−7	−32
		1000	−47	−140	−174	−3	−19	−25	−3	−15	−95	−2	−10	−31
	0.9	200	−91	−275	−293	−16	−44	−47	−27	−9	−138	−11	−18	−62
		500	−96	−251	−305	−18	−68	−65	−1	−24	−135	−5	−19	−63
		1000	−107	−256	−299	−7	−45	−46	−0	−17	−129	−4	−22	−65
Test	0.5	200	−38	−23	−70	−2	−8	−15	−4	−43	−49	−4	−1	−3
		500	−17	−5	−34	−16	−12	−21	−8	−20	−41	−5	0	−3
		1000	−18	−21	−16	−0	−1	−4	−2	−8	−29	−3	−2	−1
	0.9	200	−6	−36	−28	−11	−22	−25	−31	−19	−57	−8	−4	−19
		500	−3	−42	−63	−5	−27	−27	0	−13	−44	−3	−5	−19
		1000	−24	−61	−75	−4	−2	0	−2	−8	−49	−2	−11	−30

Table 10: MSE (× 1000) of SEM and test-based estimator (based on LRT) under MNAR-Y.

			P_x											
			0.3						0.8					
			P_y						P_y					
			0.3			0.8			0.3			0.8		
Methods	$\rho_{M_x^* Y}$	N	$\widehat{\beta}_X$	$\widehat{\beta}_Z$	$\widehat{\psi}_{XZ}$	$\widehat{\beta}_X$	$\widehat{\beta}_Z$	$\widehat{\psi}_{XZ}$	$\widehat{\beta}_X$	$\widehat{\beta}_Z$	$\widehat{\psi}_{XZ}$	$\widehat{\beta}_X$	$\widehat{\beta}_Z$	$\widehat{\psi}_{XZ}$
SEM	0.5	200	32	60	61	14	30	24	31	33	18	10	13	9
		500	13	30	38	7	10	7	8	16	10	4	5	4
		1000	8	28	35	3	5	5	5	6	10	1	2	2
	0.9	200	64	127	112	19	36	32	29	35	27	8	13	10
		500	29	86	103	8	18	14	6	16	21	4	5	7
		1000	22	80	94	3	8	6	4	7	18	1	2	5
Test	0.5	200	32	57	52	13	30	24	32	33	23	10	13	10
		500	13	23	19	7	10	6	8	16	9	4	5	3
		1000	5	12	9	3	4	4	5	6	5	1	2	1
	0.9	200	44	64	52	18	36	32	27	34	22	8	13	9
		500	15	27	25	8	13	9	6	15	10	3	4	4
		1000	11	27	27	2	6	4	3	7	8	1	2	3

Table 11: Bias (× 1000) of SEM and test-based estimator (based on LRT) under R-MAR.

		P_x											
		0.3						0.8					
		P_y						P_y					
		0.3			0.8			0.3			0.8		
Methods	N	$\widehat{\beta}_X$	$\widehat{\beta}_Z$	$\widehat{\psi}_{XZ}$	$\widehat{\beta}_X$	$\widehat{\beta}_Z$	$\widehat{\psi}_{XZ}$	$\widehat{\beta}_X$	$\widehat{\beta}_Z$	$\widehat{\psi}_{XZ}$	$\widehat{\beta}_X$	$\widehat{\beta}_Z$	$\widehat{\psi}_{XZ}$
SEM	200	−5	−12	−4	29	−22	−15	−12	−12	−14	−5	−0	−0
	500	−7	−10	−7	17	−8	−5	−18	−16	−7	−4	−2	−4
	1000	−9	−2	−4	0	−4	−3	−8	−9	−1	−2	−4	0
Test	200	−10	−13	−8	22	−10	−21	−13	−13	−7	−5	−0	−2
	500	−11	−16	−4	17	−7	−3	−18	−17	−10	−4	−2	−4
	1000	−9	−3	−5	0	−3	−3	−8	−9	−3	−2	−3	0

Table 12: MSE (× 1000) of SEM and test-based estimator (based on LRT) under R-MAR.

		P_x											
		0.3						0.8					
		P_y						P_y					
		0.3			0.8			0.3			0.8		
Methods	N	$\widehat{\beta}_X$	$\widehat{\beta}_Z$	$\widehat{\psi}_{XZ}$	$\widehat{\beta}_X$	$\widehat{\beta}_Z$	$\widehat{\psi}_{XZ}$	$\widehat{\beta}_X$	$\widehat{\beta}_Z$	$\widehat{\psi}_{XZ}$	$\widehat{\beta}_X$	$\widehat{\beta}_Z$	$\widehat{\psi}_{XZ}$
SEM	200	36	51	27	21	24	15	28	30	10	9	12	6
	500	13	19	7	6	8	6	12	12	3	3	3	3
	1000	7	11	4	3	4	3	3	6	1	2	2	1
Test	200	40	68	28	27	29	18	28	30	17	9	12	7
	500	15	23	8	6	9	6	12	12	4	2	3	3
	1000	7	11	4	3	4	3	3	6	1	2	2	1
